# Effect of Maternal Dietary DHA and Prenatal Stress Mouse Model on Autistic-like Behaviors, Lipid Peroxidation Activity, and GABA Expression in Offspring Pups

**DOI:** 10.3390/ijms26146730

**Published:** 2025-07-14

**Authors:** Taeseon Woo, Nick I. Ahmed, Michael K. Appenteng, Candice King, Runting Li, Kevin L. Fritsche, Grace Y. Sun, Jiankun Cui, Matthew J. Will, Sara V. Maurer, Hanna E. Stevens, David Q. Beversdorf, C. Michael Greenlief

**Affiliations:** 1 Interdisciplinary Neuroscience Program, University of Missouri, Columbia, MO 65211, USA; tw2954@nyu.edu; 2 Department of Psychological Sciences, University of Missouri, Columbia, MO 65211, USA; niac86@missouri.edu (N.I.A.); willm@missouri.edu (M.J.W.); 3 Department of Chemistry, University of Missouri, Columbia, MO 65211, USA; mk-appenteng@bethel.edu; 4 Department of Biological Science, University of Missouri, Columbia, MO 65211, USA; cking86@gmail.com; 5 Department of Pathology and Anatomical Sciences, University of Missouri School of Medicine, Columbia, MO 65212, USA; lirun@health.missouri.edu (R.L.); cuij@health.missouri.edu (J.C.); 6 Department of Nutrition and Exercise Physiology, University of Missouri School of Medicine, Columbia, MO 65212, USA; klfritsche37@gmail.com; 7 Department of Biochemistry, University of Missouri, Columbia, MO 65211, USA; sung@health.missouri.edu; 8 Department of Psychiatry, Carver College of Medicine, University of Iowa, Iowa City, IA 52242, USA; sara.maurer.phd@gmail.com (S.V.M.); hanna-stevens@uiowa.edu (H.E.S.); 9 Department of Radiology, Neurology, and Psychological Science, William and Nancy Thompson Endowed Chair in Radiology, University of Missouri, Columbia, MO 65211, USA

**Keywords:** autism spectrum disorder, prenatal stress, GABA, docosahexaenoic acid (DHA), serotonin transporter

## Abstract

Autism spectrum disorder (ASD) is a neurodevelopmental disorder characterized by restricted social communication and repetitive behaviors. Prenatal stress is critical in neurodevelopment and increases risk for ASD, particularly in those with greater genetic susceptibility to stress. Docosahexaenoic acid (DHA) is one of the most abundant ω-3 fatty acids in the membrane phospholipids of the mammalian brain, and dietary DHA plays an important role in brain development and maintenance of brain structure. In this study, we investigated whether peri-natal supplementation of DHA can alleviate autistic-like behaviors in a genetic risk/stress mouse model and how it alters lipid peroxidation activity and GABAergic system gene expression in the forebrain. Pregnant heterozygous serotonin transporter knockout (SERT-KO) and wild-type (WT) dams were placed in either non-stressed control conditions or chronic variable stress (CVS) conditions and fed either a control diet or a DHA-rich (1% by weight) diet. Offspring of each group were assessed for anxiety and autism-associated behavior at post-natal day 60 using an open field test, elevated plus maze test, repetitive behavior, and the 3-chamber social approach test. A liquid chromatography-mass spectrometry (LC-MS)-based method was used to follow changes in levels of lipid peroxidation products in the cerebral cortex. Male offspring of prenatally stressed SERT-het KO dams exhibited decreased social preference behaviors and increased repetitive grooming behaviors compared to WT control offspring. Moreover, DHA supplementation in male SERT-het mice decreased frequency of grooming behaviors albeit showing no associated effects on social behaviors. Regardless of stress conditions, supplementation of DHA to the WT mice did not result in alterations in grooming nor social interaction in the offspring. Furthermore, no apparent changes were observed in the lipid peroxidation products comparing the stressed and non-stressed brains. *Gad2* was downregulated in the cortex of female offspring of prenatally stressed SERT-KO dams, and this change appeared to be rescued by DHA supplementation in offspring. *Gad2* was upregulated in the striatum of male offspring of prenatally stressed SERT-KO dams, but DHA did not significantly alter the expression compared to the control diet condition.

## 1. Introduction

Autism spectrum disorder (ASD) is a neurodevelopmental disorder characterized by restricted social communication and repetitive interests and behaviors [1,2,3,4,5]. Autism is among the most enigmatic disorders of child development, with a prevalence of about 1 in 31 among 8-year-old children in the United States according to estimates from the CDC collected in 2022 [6]. Globally, prevalence varies widely, and a systematic review of 71 studies published from 2012 to 2021 across 34 countries estimated a median prevalence of 1 in 100 children with ASD [7]. Direct medical, non-direct medical, and productivity combined costs per year in the US were estimated to be USD 268 billion in 2015, and the economic burden is forecasted to rise to USD 461 billion by 2025 [8]. Numerous studies have associated prenatal maternal stress with a risk of neurodevelopmental disorders in the offspring [9,10,11,12,13,14,15]. A study by Breen et al. [16] indicated that prenatal exposure to maternal psychological distress could induce neuronal, immunological and behavioral abnormalities in affected offspring. Several studies have also suggested that prenatal stress is a possible risk factor in the development of autism spectrum disorders [17,18,19,20,21]. However, many children exposed to stress prenatally are born healthy and develop normally, suggesting that other factors may contribute to autism in this setting [22]. Genes that contribute to stress reactivity may, therefore, exacerbate prenatal stress-mediated behavioral changes in offspring. One candidate gene linked to increased stress reactivity is the serotonin transporter gene [23,24,25]. In humans, the short allele polymorphism of the serotonin transporter gene was linked to ASD in some studies [24,26]. In animal models, the heterozygous serotonin transporter (SERT) knockout mouse with reduced serotonin uptake activity and transporter binding availability [27] is similar to that seen in the human short allele carriers. In an earlier study by Beversdorf and co-workers, they looked at the combined effect of maternal serotonin transporter genotype and prenatal stress in modulating offspring social interaction in mice [1]. Data from this study indicated a possible combined effect of maternal serotonin transporter genotype and prenatal stress contributing to the production of autistic-like behavior in offspring. In another study, the Beversdorf’s group examined a maternal genetic variation in the promoter region of the serotonin transporter gene (5-HTTLPR) on stress tolerance and its interaction with the effect of environmental stressors on ASD risk in a clinical population. Mothers of ASD children reported incidents of stress and severity during the pregnancy along with whole blood samples to identify short and long 5-HTTLPR polymorphisms. Carrying of the short allele was related to the number of prenatal stress exposures reported during the critical gestational periods of their ASD children, but this effect was nonexistent when the same mothers recalled pregnancies of neurotypical siblings. This work concurred with previous findings that illustrated temporal and genotypic specificity in human development of ASD [17,28].

Docosahexaenoic acid (DHA, C22:6, n-3), is an omega-3 polyunsaturated fatty acid (PUFA) enriched in membrane phospholipids in the brain [29]. Recent studies have demonstrated the pleiotropic properties [30] of DHA with diverse health effects [31,32,33]. While DHA is important for adequate brain development and cognition [34,35,36], its deficiency is associated with impaired visual, attention, and cognition deficits, precipitation of psychiatric symptoms, and increased vulnerability to neuronal atrophy [37,38,39,40,41]. Other studies have found that DHA may have an anti-stress function [42,43,44] and can be converted to oxylipins, which regulate cell redox homeostasis and contribute to antioxidant pathways [29]. A study by Tang et al. [45] examining the mental health of weaning female rats found that altered dopamine or norepinephrine transmission in the brain may be a key neuronal mechanism that contributes to the potential detrimental effects of maternal dietary n-3 PUFA deficiency. While some studies [46,47] have claimed that supplementation of ω-3 long chain PUFA could improve social interaction and repetitive and restricted interests, other studies [48,49,50,51,52,53] have been inconclusive in suggesting the effects of ω-3 PUFA supplementation on ASD. Nevertheless, an earlier study by Beversdorf’s group indicated that autism associated behaviors and changes in the dopaminergic system in offspring mice exposed to prenatal stress could be mitigated through maternal DHA supplementation [47]. A related study by Feng et al. [54] found that maternal feeding of DHA could exert preventive effects on prenatal stress-induced brain dysfunction and that modulation of mitochondrial metabolism might play a critical role in DHA protection. A study by Gao et al. [55] showed that maternal DHA supplementation protected rat offspring against learning and memory impairment following prenatal exposure to valproic acid. Another study by Kunio et al. [56] also suggested that supplementation of DHA with arachidonic acid (ARA), an (n-6)PUFA, could improve social interaction in individuals with ASD by up-regulating signal transduction. Nevertheless, despite differences in the metabolism of DHA and ARA through an apparent Yin–Yang mechanism [57], both PUFAs can undergo peroxidation through enzymatic and non-enzymatic processes, yielding 4-hydoxyhexenal (4-HHE) for DHA and 4-hydroxynonenal (4-HNE) for ARA [58]. These peroxidation products have been used to represent metabolism of the respective PUFA under different conditions. In a recent study [59], a maternal DHA-enriched diet was shown to result in an increase in DHA and decrease in ARA in the pup brain. In this study, the increase in brain DHA was linked to an increase in 4-HHE, although no change was observed in 4-HNE [59,60]. However, the role of maternal dietary DHA in a gene/stress mouse model, and how it may affect lipid peroxidation activity and autistic-like behaviors in offspring remains to be investigated.

Maternal SERT variation may also have implications for early fetal forebrain development. Prior to the development of endogenous sources of 5-HT in the fetus, previous work in a *Pet-1^−/−^* murine model, lacking 5-HT in the dorsal raphe nucleus, aided in identifying the placenta itself and not maternal blood moving through the placenta as the key exogenous origin of 5-HT needed for fetal brain development [61,62,63]. Interestingly, the maternal short and long allele variants of SERT are related to 5-HTT transcription levels in the placenta, with those with S/L and S/S showing similarly reduced levels of 5-HTT and decreased uptake of H^3^-5-HT compared to the L/L group [64]. Recent work has suggested that in male rats, independent of their 5-HTT^+/−^ genotypic mother, the offspring’s own 5-HTT genotype increased anxiety and depressive-like behaviors along with possible alterations in GAD67 and parvalbumin mRNA levels in the infralimbic cortices [65]. In concert with this maternal–fetal genotype interaction, it is also important to consider the impact of environmental interaction, particularly in the context of prenatal stress exposure.

Animal models of prenatal stress exposure have repeatedly recapitulated characteristic molecular deficits identified in neurodevelopmental disorders. As ASD has previously been shown to involve the dysregulation of the excitatory/inhibitory balance, of particular interest are components within the GABAergic (inhibitory) system. GABA is synthesized via the rate-limiting enzyme glutamate decarboxylase (GAD) from isoforms GAD 67 (coded by *Gad1*) and GAD 65 (*Gad2*) and acts as an inhibitory neurotransmitter for mature neurons in the brain [66,67,68]. These isoforms were reduced in postmortem brain samples of autistic subjects. Furthermore, prenatal stress in mice alters GAD expressing interneurons in cerebral cortex and GABAergic projection neurons in the striatum, during development, and in the mature brain [69,70,71]. The striatum’s interneurons are primarily inhibitory and largely express parvalbumin (PV+), while the cerebral cortex is composed of up to 40% PV+ interneurons. Prenatal stress can reduce the subpopulations of PV+ neurons in the cerebral cortex of mice with already dysregulated Gad1 expression [72]. Previous work by co-author Stevens also showed how prenatal stress arrests GABAergic system development and maintains lower populations of the parvalbumin subtype [73,74]. In comparison, somatostatin (SST) + interneurons make up only 5–10% of striatal interneurons and close to 30% of cortical interneurons. SST+ dysregulation has not been as well studied as PV in the context of prenatal stress, but dysregulation of SST interneurons has been connected to ASD and related to stereotypic behaviors [75] and social deficits [76]. While prenatal stress has not been studied for striatal interneuron changes, striatal monoamine neurotransmitters are disrupted by prenatal stress. Given the importance of the GABAergic system in ASD, Gad2 expression is examined in this study as it is also critical in GABA synthesis in order to gain a more complete picture of the effects on the GABAergic system [66,67,68]. Work done earlier by this group assessed the effects of maternal supplementation of DHA on the offspring of stressed SERT-KO dams and found both dopamine and 3,4-dihydroxyphenylacetic (DOPAC) to be rescued from elevated levels in the striatum compared to the non-supplemented gene/stress model counterpart, but found no differences in 5-HT [47]. While we are not proposing a direct relationship between DHA and the GABAergic system, the GABAergic system in this study serves as a molecular marker for the mitigation of the effects of prenatal stress by DHA, given the established relationship between prenatal stress and impacts on the GABAergic system [73,74].

In this study, pregnant heterozygous serotonin transporter knockout-SERT-KO (HT) and wild-type (WT) mice were fed either a control diet or a DHA-rich (1% by wt) diet and placed in either a non-stressed control condition or a chronic variable stress condition. Male and female offspring from each group were assessed for anxiety and autism-associated behavior at post-natal day 60, including an open field test, elevated plus maze test, repetitive behavior, and the 3-chamber social approach test. Investigations were carried out to ascertain whether prenatal supplementation of DHA could alleviate autistic-like behaviors in a gene/stress mouse model, ASD-related GABA expression, and lipid peroxidation activity in the brain.

## 2. Results

### 2.1. Offspring Behavioral Results

#### 2.1.1. Social Interaction (Single Stranger Versus Novel Object)

**Figure 1:**
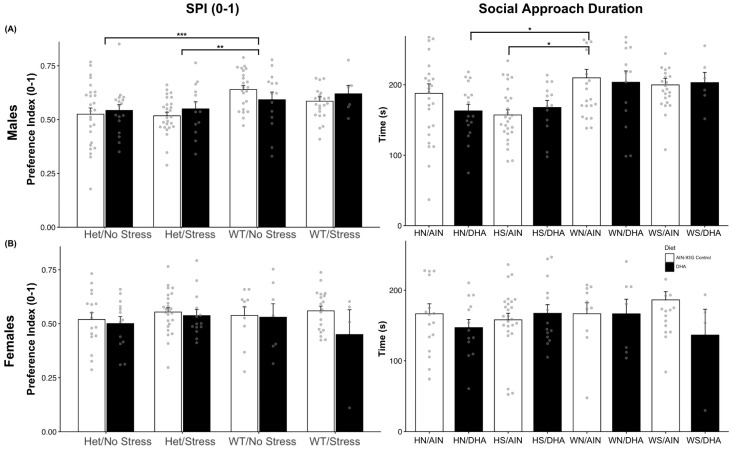
(**A**) Two-way ANOVA of SPI (left) in males showed a main effect of gene × stress mouse model, driven by non-stressed WT mice exhibiting higher SPI scores than both non-stressed SERT-het males (*p* < 0.001 and stressed SERT-het males (*p* < 0.01) regardless of diet. Duration (right) interacting with the novel stranger was significantly lower in control-fed HS males and DHA-fed HN males than control-fed WN males. (**B**) Females did not show any differences in SPI (left) or in the duration of time (right) spent investigating the novel stranger across all groups and diets.* *p* < 0.05; ** *p* < 0.01; *** *p* < 0.001.

Social interaction measures were assessed in 142 male mice ((HS/C (n = 27), HS/D (n = 13), HN/C (n = 26), HN/D (n = 17), WS/C (n = 23), WS/D (n = 6), WN/C (n = 23), WN/D (n = 14)) and 109 females ((HS/C (n = 25), HS/D (n = 14), HN/C (n = 17), HN/D (n = 13), WS/C (n = 19), WS/D (n = 4), WN/C (n = 10), WN/D (n = 7)). The proportion of time spent with the novel stranger compared to the empty cup, the social preference index (SPI), was calculated as time spent proximal to the novel stranger divided by the time spent proximal to the novel stranger or proximal the opposite chamber’s empty cup. A two-way ANOVA (mouse model × diet) was conducted on SPI as well as on the total duration an experimental mouse was proximal to the novel stranger. All male and female (except for 1 group, WS/D) mice showed a preference for the novel stranger mouse over the novel object (SPI > 0.50), but SPI only showed a significant main effect across mouse models, F(3, 134) = 5.92, *p* < 0.001, in males (Figure 1A). SERT-het males prenatally stressed (M = 0.54, SE = 0.01, n = 38, *p* = 0.0025) and not stressed (M = 0.53, SE = 0.02, n = 40, *p* < 0.001) scored lower on SPI than did non-stressed wild-type males (M = 0.62, SE = 0.02, n = 36). This can be further elucidated using the total duration of social interaction. A subsequent two-way ANOVA pointed primarily to a main effect of mouse model (*p* = 0.01) and possibly diet (*p* = 0.054). Supporting a priori hypotheses, post hoc analysis indicated that control diet samples of HS (M = 161.35, SE = 7.24) spent less total time in proximity to the novel stranger than the control diet WN mice (M = 203.80, SE = 10.90; *p* < 0.05), but DHA-supplemented HS (M = 167.78, SE = 10.10; *p* = 0.30) males did not spend significantly less time than WN/AIN males (Figure 1A). Contrary to males, social preference behavior did not differ amongst female mice (Figure 1B). Female mice did not show a main effect across mouse models (F(3, 101) = 0.41, *p* = 0.745), diet (*p* = 0.669), or their interaction (*p* = 0.616) on SPI. This extends to the analysis of duration spent with the novel stranger, as they did not significantly differ by mouse model (F(3, 101) = 1.17, *p* = 0.325) or by DHA supplementation (F(1, 101) = 1.11, *p* = 0.294).

#### 2.1.2. Social Novelty Interaction (Novel Stranger Versus Familiar Stranger)

**Figure 2:**
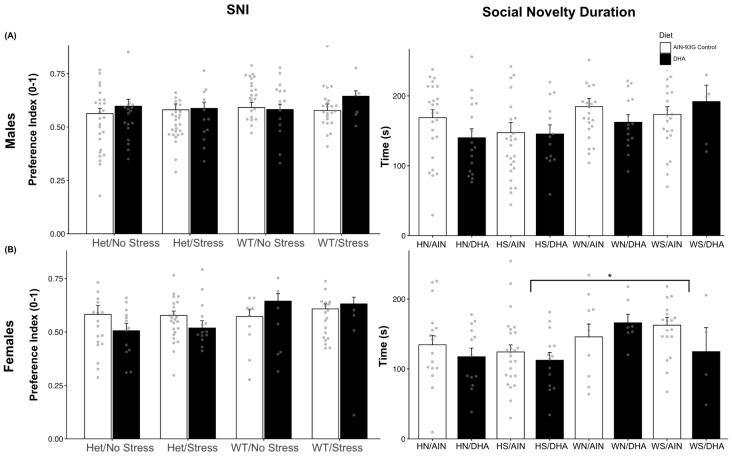
(**A**) No significant differences were found between SNI scores (left) in males or in the durations (right) spent with the novel stranger in the social novelty phase. (**B**) Females also did not show any significant differences in SNI (left); however, HS females (across diets) displayed less chamber time (right) with the novel stranger when compared to WS females (F(3,101) = 3.36, *p* < 0.05). HS-born females (M = 119.98, SE = 7.56, n = 39) spent less time interacting with the novel stranger than did WS-born females (M = 155.9565, SE = 10.8528, n = 23) (*p* = 0.0342), and a similar trend was emerging when compared to WN-born females(M = 154.1235, SE = 11.851) (*p* = 0.090). * *p* < 0.05.

Social novelty indices (SNI) are a measure of social recognition and novelty preference in mice. Previous ASD models have exhibited lower or no preference for social novelty (SNI ≤ 0.50), exhibiting social memory for the familiar stranger and an aversion to novel social partners [74,77,78]. All male offspring exhibited a preference for the novel stranger (SNI > 0.50), and a two-way ANOVA of these scores showed that neither model (*p* = 0.492) nor diet (*p* = 0.348) resulted in differences in SNI (Figure 2A). The total duration spent with the novel stranger did not differ across models, F(3, 134) = 1.33, *p* = 0.267, but DHA supplementation trended towards a possible main effect, F(1, 134) = 3.44, *p* = 0.07. Female offspring revealed similar results, as there was no main effect of mouse model on SNI, F(3, 101) = 0.3, *p* = 0.824, as all displayed preference for the novel stranger, but diet may display a trend, F(1, 101) = 2.98, *p* = 0.087, despite no interaction effects, F(3, 101) = 1.75, *p* = 0.162 (Figure 2B). Parsing these indices by their durations, female offspring did not differ in the time spent with their “familiar” stranger (F(3, 101) = 1.23, *p* = 0.304) but showed a significant effect of the model on the time spent with the novel stranger (F(3,101) = 3.36, *p* < 0.05). HS-born females (M = 119.98, SE = 7.56, n = 39) spent less time interacting with the novel stranger than did WS-born females (M = 155.9565, SE = 10.8528, n = 23) (*p* = 0.0342), and a similar trend emerged when compared to WN-born females (M = 154.1235, SE = 11.851) (*p* = 0.090). Females from the control diet litters HS = 124.232, SE = 10.0981, n = 25; HN= 134.5353, SE = 13.5462, n = 17; WS = 162.5263, SE = 10.9141, n = 19), except for those of the WN background, spent more time than did their DHA-treated counterparts (HS = 112.3786, SE = 11.069, n = 14; HN = 117.3846, SE = 12.3206, n = 13; WS, D = 124.75, SE = 34.3326, n = 4) with the novel stranger. Only in the WN background did DHA seem to raise (M = 165.9571, SE = 12.2202, n = 7) the novel interaction time over the control diet (M = 145.84, SE = 18.3373, n = 10).

#### 2.1.3. Grooming Behaviors

**Figure 3:**
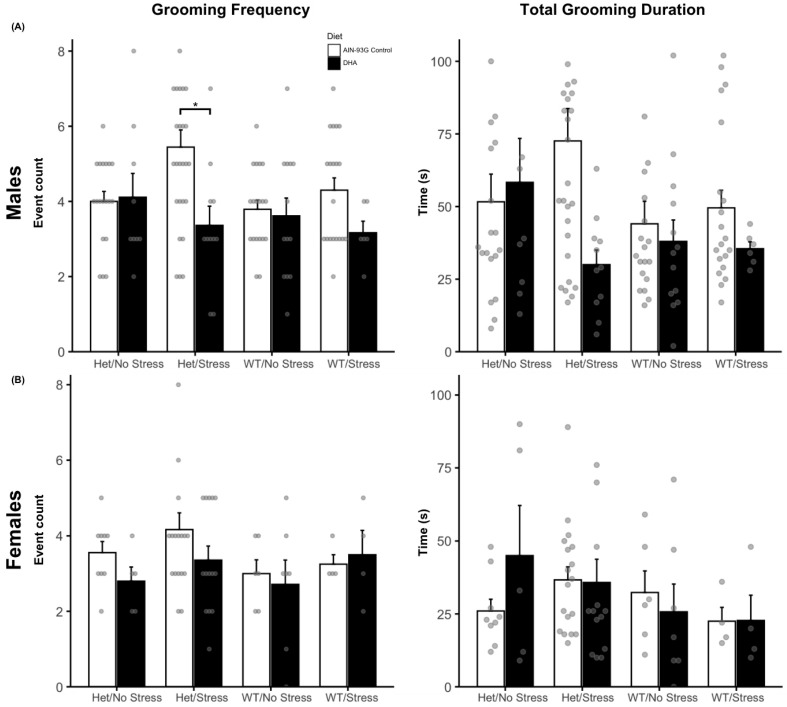
(**A**) Grooming frequency of males was highest in control-fed HS males, but grooming decreased in frequency (left) and nearly duration (right) in DHA-supplemented HS males (*p* = 0.10). (**B**) Females did not show any differences in grooming frequency (left) or duration (right) across mouse models and diet. * *p* < 0.05.

Spontaneous grooming behavior was assessed in 124 male (WN/C (n = 19); WN/D (n = 13); WS/C (n = 19); WS/D (n = 6); HN/C (n = 17); HN/D (n = 9); HS/C (n = 25); HS/D (n = 11)) and 67 female mice (WN/C (n = 6); WN/D (n = 7); WS/C (n = 4); WS/D (n = 4); HN/C (n = 9); HN/D (n = 5); HS/C (n = 18); HS/D (n = 14)) by frequency and duration of grooming events. In male mice, a two-way ANOVA showed a significant main effect of the mouse model for grooming frequency (F(3, 111) = 4.06, *p* = 0.009), and a possible interaction effect (*p* = 0.07) but no main effect of the diet (*p* = 0.94). Post hoc tests confirmed that the WN group exhibited a lower grooming frequency (M = 3.72, SE = 0.22) compared to the HS group (M = 4.81, SE = 0.40, *p* = 0.0469) averaged across diets (Figure 3A). Additionally, a priori driven *t*-tests found that DHA-supplemented HS mice exhibited decreased grooming (M = 3.36, SE = 0.51, *p* < 0.05) compared to their control diet HS counterparts (M = 5.44, SE = 0.49). The total grooming duration was then compared and did not show any significant main effects (Model: *p* = 0.15; Diet: *p* = 0.67) or interactions (*p* = 0.16). As hypothesized, HS control diet males spent the most time grooming (M = 69.92 s, SE = 11.57) descriptively, but this was not more than the grooming duration of control-fed WN males (M = 44.1, SE = 7.74, *p* = 0.38). DHA-supplemented HS males did show a nearly significant decrease in duration (M = 30 s, SE = 5.03, *p* = 0.10) compared to control-fed HS males and spent the least total time grooming than all other mice (Figure 3A). This pattern aligns with the grooming frequency results, further suggesting a potential interaction among the genetic background, stress exposure, and diet that may warrant further investigation. For female mice, no significant main effects or interactions were observed for either grooming frequency (Model: *p* = 0.288; Diet: *p* = 0.346; interaction: *p* = 0.785) or grooming duration (Model: *p* = 0.57; Diet: *p* = 0.144; interaction: *p* = 0.51; see Figure 3B).

#### 2.1.4. Marble Burying

Marble burying (MB) behaviors were recorded for 143 male mice ((WN/C (n = 22); WN/D (n = 14); WS/C (n = 22); WS/D (n = 6); HN/C (n = 24); HN/D (n = 13); HS/C (n = 25); HS/D (n = 11)) and 98 female mice (WN/C (n = 10); WN/D (n = 3); WS/C (n = 19); WS/D (n = 4); HN/C (n = 17); HN/D (n = 13); HS/C (n = 24); HS/D (n = 11)). Male mice showed a significant interaction between the mouse model and diet (F(3, 128) = 2.95, *p* = 0.035, and post hoc analysis revealed this interaction to be primarily driven by SERT-het males not prenatally stressed, as they buried fewer marbles when treated with DHA (M = 3.46, SE = 1.04, *p* = 0.01) than control supplementation (M = 9.33, SE = 1.12). HS males did not differ in marble burying behavior when compared to wild-type controls regardless of diet (*p* > 0.05). No differences were detected in female marble burying behaviors (Model: *p* = 0.755; Diet: *p* = 0.18) or the interaction (*p* = 0.54).

#### 2.1.5. Open Field

Open field behaviors were measured in 124 male mice (WN/C (n = 17); WN/D (n = 6); WS/C (n = 19); WS/D (n = 6); HN/C (n = 17); HN/D (n = 16); HS/C (n = 25); HS/D (n = 11)) and 94 female mice (WN/C (n = 6); WN/D (n = 5); WS/C (n = 16); WS/D (n = 4); HN, C (n = 16); HN, D (n = 13); HS, C (n = 20); HS, D (n = 14)). Both males and females showed no differences in distance travelled in the open field across all mouse models (*p* = 0.092; *p* = 0.361), regardless of DHA supplementation (*p* = 0.165; *p* = 0.227). Likewise, time spent in the inner region of the open field did not differ by model or diet supplementation in males (*p* = 0.465; *p* = 0.673) or females (*p* = 0.460; *p* = 0.229). By model and by diet, males and females did not appear to differ in locomotor activity or exploratory behaviors in the open field.

#### 2.1.6. Elevated Plus Maze Results

**Figure 4:**
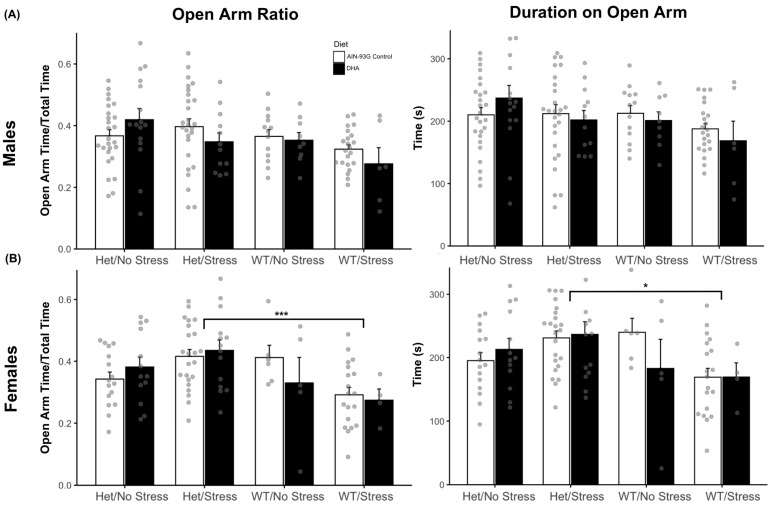
Open arm ratio (OAR) and time spent on open arms were analyzed. (**A**) Males showed no significant differences across models or diets. (**B**) Females exhibited a main effect of the mouse model on OAR (F(3, 92) = 4.94, *p* = 0.003). Stressed SERT-het (HS) females had significantly higher OAR (M = 0.423, SE = 0.02) compared to stressed wild-type (WS) females (M = 0.29, SE = 0.02, *p* < 0.001). HS females also spent more time on open arms regardless of diet (Control: M = 230.95, SE = 10.9; DHA: M = 236.49, SE = 20.1) compared to WS females (M = 169.10, SE = 11.98, *p* < 0.05). WN = non-stressed wild type, WS = stressed wild type, HN = non-stressed SERT-het, HS = stressed SERT-het Data are presented as the mean ± SEM. * *p* < 0.05, *** *p* < 0.001.

Elevated plus maze (EPM) can be scored using the open arm ratio (OA ratio), which quantifies the proportion of time an experimental mouse spends on the open arm of the apparatus, indicating decreased anxiety, compared to the total duration of the test. Two-way ANOVAs were run on the OA ratio, as well as the specific durations of time spent on either arm of the EPM. In males, there were no main effects of mouse model (*p* = 0.24), diet (*p* = 0.09), or interaction (*p* = 0.16) on the OA ratio (Figure 4A). Females, in contrast, did show a significant main effect of the mouse model on OA ratio scores (F(3, 92) = 4.94, *p* = 0.003). Post-hoc testing found that HS female mice (M = 0.423, SE = 0.02) had significantly higher OA ratios than WS mice (M = 0.29, SE = 0.02, *p* < 0.001, Figure 4B). Furthermore, the total duration spent on the open arm displayed the same main effect of the mouse model, as HS females, born from both control (M = 230.95, SE = 10.9, *p* < 0.05) and DHA diet groups (M = 236.49, SE = 20.1, *p* < 0.05), spent significantly more time on the open arm than did WS females (M = 169.10, SE = 11.98, Figure 4B). This may suggest that prenatal stress differently impacts SERT-het female offspring than it does wild-type females.

### 2.2. HHE and 4-HNE Levels

**Figure 5:**
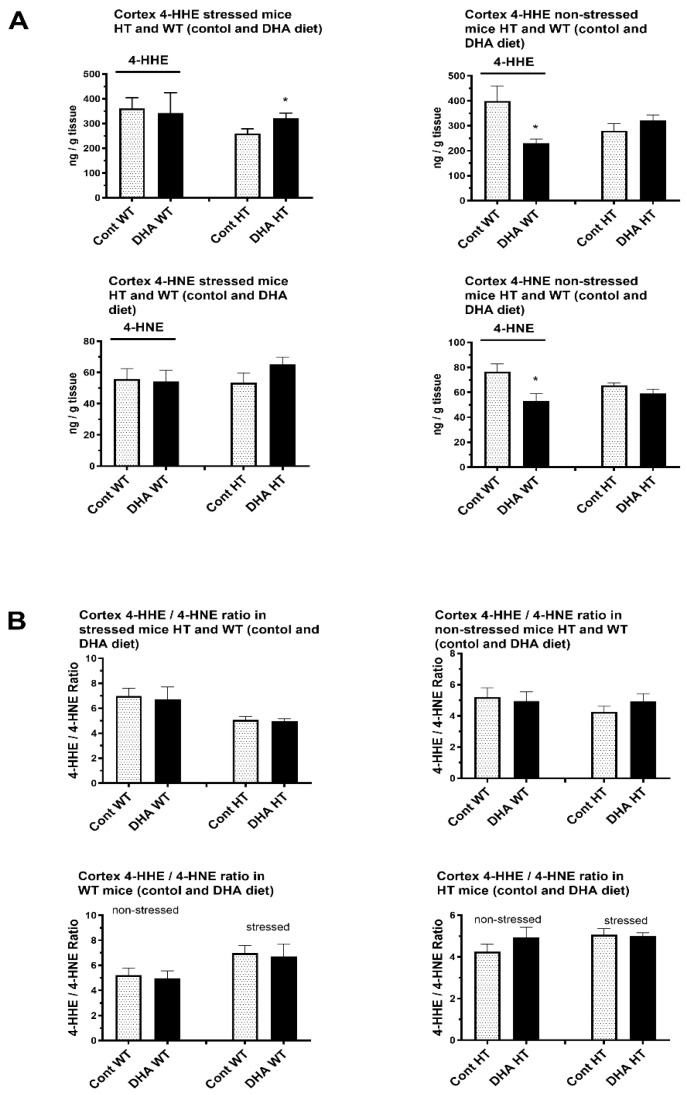
Levels of (**A**) 4-HHE, 4-HNE, and (**B**) 4-HHE/4-HNE ratio of offspring left cortex from pregnant heterozygous serotonin transporter knockout-SERT-KO (HT) and wild-type (WT) dams placed in either non-stressed control conditions or chronic variable stress conditions and fed either the control diet or DHA-rich (1% by wt.) diet. Protocol for LC-MS/MS determination of 4-HHE and 4-HNE are described in the text. Data were normalized to tissue weight. Results represent the mean ± SEM of control (n = 4) and DHA (n = 4) samples. Analysis using two-tail unpaired *t*-test indicated significance between DHA group and controls. * *p* < 0.05.

No changes in 4-HHE levels were observed for control and groups with 1% DHA-enriched diets for both the stressed (359.0 ± 0.2 ng/g; 358.8 ± 0.3 ng/g) and non-stressed (360.3 ± 0.5 ng/g; 360.4 ± 0.4 ng/g) and HT and stressed (359.3 ± 0.5 ng/g; 359.2 ± 0.5 ng/g) and non-stressed (360.7 ± 0.4 ng/g; 360.7 ± 0.4 ng/g) WT groups in the cortex (Figure 5A). A significant increase in 4-HHE levels for the control and 1% DHA groups was observed for the stressed HT group (259.9 ± 57.0 ng/g; 321.0 ± 74.9 ng/g) with no apparent change in the non-stressed HT group (288.0 ± 97.6 ng/g; 278.4 ± 68.1 ng/g). No apparent change was recorded for the stressed WT group (362.1 ± 43.9 ng/g; 343.2 ± 34.9 ng/g). The 4-HHE levels for the DHA-enriched diet for the non-stressed WT group (394.8 ± 28.0 ng/g; 230.1 ± 57.0 ng/g) showed a significant decrease compared to control diet group (Figure 5A).

Supplementation with a DHA-enriched diet resulted in a significant decrease in 4-HNE (*p* < 0.05) for the non-stressed WT group (76.5v ± 22.2 ng/g; 53.2 ± 20.9 ng/g) and no apparent change in 4-HNE for the stressed WT group (55.9 ± 23.2 ng/g; 54.3 ± 24.4 ng/g) (Figure 5A). On the other hand, the levels of 4-HNE were increased for the DHA-enriched diet in stressed HT mice (53.5 ± 18.0 ng/g; 65.1 ± 16.5 ng/g), while a slight decrease was observed for the non-stressed HT mice (65.6 ± 7.0 ng/g; 59.0 ± 12.3 ng/g) (Figure 5A).

No significant changes in the 4-HHE/4-HNE ratios were observed. A slight increase in the ratio for the dietary DHA supplement in the non-stressed HT group (4.3 ± 1.3; 4.9 ± 1.7) was observed with a slight decrease in the stressed HT group (5.1 ± 0.9; 4.9 ± 0.6) (Figure 5B). A slight decrease was observed for the DHA-enriched diet in the stressed group (7.0 ± 2.1; 6.7 ± 3.5) and non-stressed WT group (5.3 ± 2.0; 4.9 ± 2.1) (Figure 5B).

### 2.3. GABAergic System Gene Expression

Expression of GABAergic system genes, which reflect general inhibitory neuron function in the cortex and striatum, were measured using RT-qPCR and delta-delta-CT methods.

#### 2.3.1. *Gad1* and *Gad2* Expression

**Figure 6:**
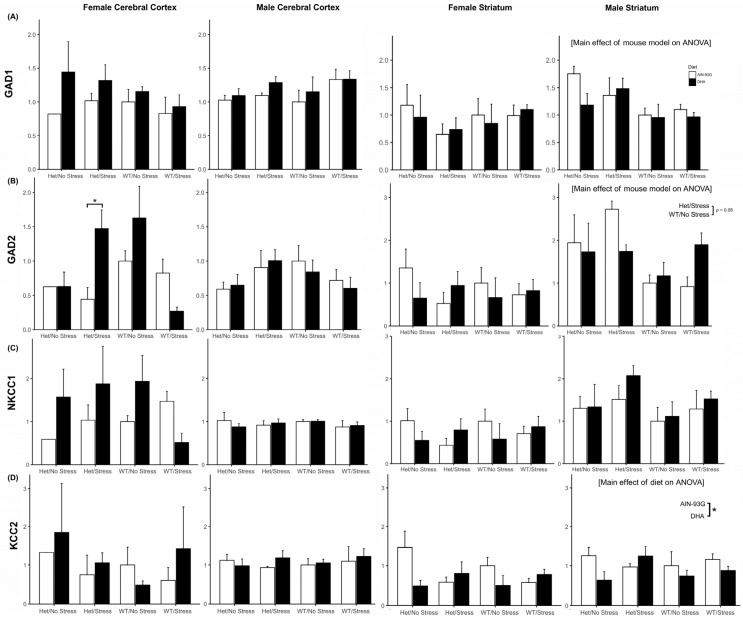
Gene expression analysis of GABA enzyme and ion transporter genes in the mouse cortex and striatum. Expression levels of (**A**) *Gad1*, (**B**) *Gad2*, (**C**) *Nkcc1*, and (**D**) *Kcc2* were measured in the female and male cortex and striatum across different mouse models and diets. Two-way ANOVAs were performed for each gene and brain region. *Gad1* expression in the male striatum showed a main effect of model (F(3, 22) = 3.35, *p* < 0.05). *Gad2* expression revealed an interaction effect between mouse model and diet in the female cortex (F(3, 22) = 5.53, *p* = 0.006) and a main effect of mouse model in the male striatum (F(3, 22) = 3.36, *p* = 0.037). *Kcc2* expression in the male striatum exhibited a main effect of diet (F(1, 22) = 5.73, *p* = 0.02). Post hoc analyses were conducted using paired *t*-tests. Significant differences (*p* < 0.05) were observed between specific groups as indicated by asterisks based on post hoc analyses of significant interaction or main effects. Main effects are identified in brackets and factor levels are compared. Interaction effects only show significant comparisons between individual groups. HS = stressed SERT-het, HN = non-stressed SERT-het, WN = non-stressed wild type. Error bars represent the standard error of the mean (SEM). n = 3–8 per group except n = 1 in female HS control diet cortex. * *p* < 0.05.

With three exceptions, expression of genes for enzymes that make GABA, *Gad1*, and *Gad2* did not differ by mouse model or diet in the cortex or striatum of either males or females (Figure 6A,B). *Gad1* in the striatum of males exhibited a main effect across mouse models (F(3, 22) = 3.35, *p* < 0.05) but was unaffected by diet (Figure 6A). Post hoc analysis showed a trend towards higher *Gad1* expression in stressed and non-stressed SERT-het males compared to non-stressed WT (*p* = 0.09, *p* = 0.07). *Gad2* expression (typically higher in expression later in the neurodevelopmental timeframe) in the cortex of females showed a different relationship, with an interaction effect of mouse model and diet (F(3, 22) = 5.53, *p* = 0.006; Figure 6B). Post hoc analysis showed that cortical *Gad2* expression was significantly higher in HS DHA-supplemented than non-supplemented HS females (*p* = 0.04). *Gad2* expression in the male striatum, similar to the male striatal *Gad1*, showed a main effect of mouse model (F(3, 22) = 3.36, *p* = 0.037) but not diet (Figure 6B). This was primarily driven by a higher expression trend in stressed SERT-het-born males (n = 7)*,* regardless of diet, as compared to the non-stressed wild types (*p* = 0.05, *n* = 8).

#### 2.3.2. Nkcc1 and Kcc2 Expression

Genes for ion transporters, *Nkcc1* and *Kcc2*, that typically control GABAergic signaling maturation changes through development, did not differ across groups in their cortical or striatal expression in males or females (Figure 6C,D) other than in the male striatum. Expression of *Kcc2* in the male striatum exhibited a main effect of diet (F(1, 22) = 5.73, *p* = 0.02), which lower with DHA supplementation across groups, with no effect of mouse model.

#### 2.3.3. Interneuron Subtype Gene Expression

**Figure 7:**
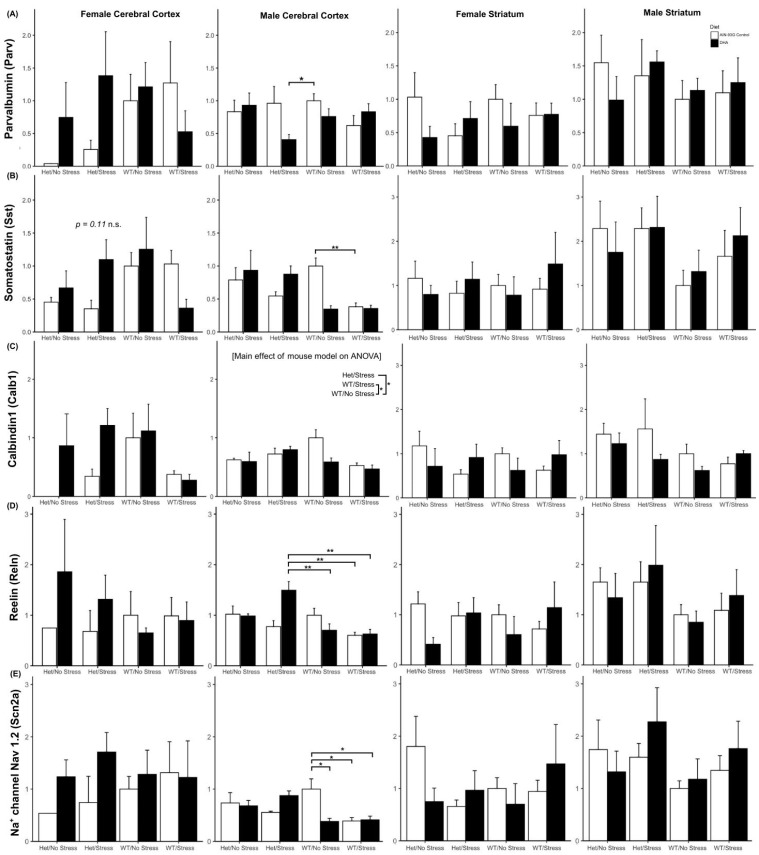
Expression analysis of GABAergic interneuron markers in the mouse cortex and striatum. Expression levels of (**A**) *Parvalbumin* (*Parv*), (**B**) *Somatostatin* (*Sst*), (**C**) *Calbindin1* (*Calb1*), (**D**) *Reelin* (*Reln*), and (**E**) *Sodium channel Nav 1.2* (*Scn2a*) were measured in the female and male cortex and striatum across different mouse models and diets. Two-way ANOVAs were performed for each gene and brain region. *Parv* expression exhibited a significant interaction between mouse model and diet (F(3, 23) = 3.31, *p* = 0.038) in the male cortex. *Sst* expression showed an interaction effect in the female cortex and significant main effects of mouse model (F(3, 23) = 5.46, *p* = 0.006) and interaction between mouse model and diet (F(3, 23) = 6.56, *p* = 0.002) in the male cortex. *Calb1* expression showed a possible main effect across mouse models in the male cortex (F(3, 23) = 2.91, *p* = 0.05). *Reln* expression was affected by a significant interaction of mouse model and diet (F(3, 23) = 4.65, *p* < 0.05). *Scn2a* expression similarly showed a significant interaction effect in the male cortex (F(3, 23) = 4.92, *p* < 0.01). Post hoc analyses were conducted using Tukey’s test and paired *t*-tests. Significant differences (*p* < 0.05) were observed between specific groups as indicated by asterisks based on post hoc analyses of significant interaction or main effects. Main effects are identified in brackets and factor levels are compared. Interaction effects only show significant comparisons between individual groups. WS = stressed wild type, WN = non-stressed wild type, HS = stressed SERT-het, HN = non-stressed SERT-het, C = control diet, D = DHA-supplemented diet. Error bars represent standard error of the mean (SEM). n = 3–8 per group. * *p* < 0.05, ** *p* < 0.01.

GABAergic interneurons include subpopulations of parvalbumin-(Parv), somatastatin-(Sst), and calbindin1-(Calb1) expressing neuronal cells, and the expression of these subtype-specific genes. A significant interaction between mouse model and diet (F(3, 23) = 3.31, *p* = 0.038) was found in *Parv* expression in the male cortex (Figure 7A). Interestingly, post hoc analysis did not find a *Parv* expression difference between the control diet groups of WN (n = 4) and HS (n = 3, *p* = 0.99), but it was significantly lower in DHA-supplemented HS (n = 4) male cortices compared to the control WN group (*p* = 0.03). No other effects were seen on *Parv* expression. Females and males both showed variation in somatostatin expression in their cerebral cortex. Similar to male cortical *Parv* expression, there was a significant interaction of mouse model and diet on *Sst* expression in the female cortex, suggesting that DHA-supplemented groups had higher expression in some het groups and lower in some WT, but no differences were significant by post hoc tests. Male cortical expression of *Sst* was altered by mouse model (main effect: F(3, 23) = 5.46, *p* = 0.006), with an interaction of mouse model and diet (F(3, 23) = 6.56, *p* = 0.002). Post hoc analysis found that control-fed WS mice were significantly lower in Sst expression than their WN/C counterparts (*p* = 0.01). *Calb1* expression in the female cortex and striatum and male striatum was not affected across groups, but expression in the male cortex showed a main effect across the mouse models (F(3, 23) = 2.91, *p* = 0.05), primarily driven by HS (*n* = 7) and WS males (*n* = 8), across diet, being lower than WN (*p* < 0.05, *n* = 8, Figure 7C).

##### Other ASD-Relevant GABAergic Genes

Expression of genes for Reelin (*Reln*) and sodium channel Nav 1.2 (*Scn2a*), GABAergic signaling genes relevant to ASD, was altered in the male cortex but had no group differences in male striatum or female cortex or striatum. In the male cortex, *Reln* expression was affected by a significant interaction of mouse model and diet (F(3, 23) = 4.65, *p* < 0.05, Figure 7D). Posthoc testing demonstrated that this was driven by increased expression of *Reln* in DHA-supplemented HS males (*n* = 4) compared to WS/A (*p* < 0.01, *n* = 4), WS/D (*p* < 0.01, *n* = 4), and WN/D (*p* < 0.01, *n* = 4) but not compared to WN/A (*p* = 0.40, *n* = 4). The increase was also nearly significantly higher than *Reln* expression in HS/A (*p* = 0.06, *n* = 3). *Scn2a* expression similarly showed a significant interaction effect in the male cortex (F(3, 23) = 4.92, *p* < 0.01), which was predominantly driven by control-fed WS males (*n* = 4), WS/D males (*n* = 4), and WN/D males (*n* = 4) expressing significantly lower *Scn2a* than the control-fed WN male cortices (*p* < 0.05, *n* = 4, Figure 7E).

## 3. Discussion

The gene × stress model of ASD produced behaviors consistent with previous generations of this ASD mouse model [1,47,79]. A decreased social preference index and duration of interaction with the stranger was exhibited in both prenatally stressed and non-stressed SERT-het males, but this was not seen in females. The addition of DHA to the diet did not rescue these social deficits in the males or females. We did not see any changes in novelty seeking in males or females regardless of the mouse model or diet. Repetitive behaviors are a key component to the diagnostic criteria for ASD and were recapitulated in this generation of prenatally stressed HS mice. The frequency of these grooming behaviors was reduced in the DHA-supplemented group of HS mice compared to control HS males, but the total duration of time was not decreased significantly. These results replicated previous effects of prenatal stress on social communication and repetitive behaviors [47], but DHA only reversed the effects of repetitive grooming events. The additional preferential effect on males in this study is of interest given the fact that ASD is considerably more prevalent in males [6], and this is also consistent with the available studies supporting a preferential effect of prenatal stress on males [80]. Therefore, this mechanism could potentially contribute to the male predominance in ASD.

While there were some limitations of the assessment here on GABAergic system gene expression, there were interesting findings in both the cortex and striatum. *Gad1* and *Gad2* expression in males was only affected in the striatum by the mouse models and not diet. Interestingly, this result is similar to recent findings of prenatal stress increasing GAD67GFP+-expressing striatal populations only in males across developmental stages [70]. In females, on the other hand, *Gad2* did show signs of reduced cortical expression in HS offspring fed the control diet and was significantly increased in the DHA-supplemented HS females. These results were similar to previous assessments showing reduced sex-specific adult cortical GAD67GFP+ cells after prenatal stress, rescued by other perinatal maternal treatments [71,73]. As with the behavioral results, the findings for *Gad1* are consistent with previous work demonstrating the effects of prenatal stress [70], but the lack of reversal of this effect with DHA supplementation suggests that behavioral effect reversal is not mediated by the effects of GABA. One might raise the question of whether the reversal of the *Gad2* findings with DHA supplementation in females could represent a potential mechanism for protection from diet in females. Further study will be needed to explore this possibility. Overall, our findings in combination with these others support sex- and region-specific effects of these stress models on the developmental trajectory of the GABAergic system; only some may be impacted by DHA diet supplementation and other neuroprotective interventions. Further research is needed to completely elucidate the origins of sex, region, and developmental differences. While investigation here of interneuron gene expression and other genes playing a role in GABAergic did not align with some previous work showing impacts of prenatal stress, there were some technical methodological limits here and differences in the stress model used. Previous work has linked behaviors, such as repetitive grooming, to the striatum, and it appears plausible that behavioral observations and relative changes in grooming behaviors may be related to male-specific striatal differences [47]. Similarly, cerebral cortex alterations could be otherwise related to social communication differences, as female and male cortices also seem to differ regarding how social approach and social novelty manifest in a sex-specific manner. A more comprehensive assessment of molecular mechanisms is critical for better understanding of the behavioral outcomes revealed in this exploratory work. Larger studies will also be needed to examine the interaction between effects, including studies with sufficient power to explore the relationship between behavioral outcomes and GABAergic outcomes.

In the present study, pregnant heterozygous serotonin transporter knockout (SERT-KO) and wild-type (WT) dams were placed in either a non-stressed control condition or in a chronic variable stress condition and fed either a control diet or DHA-supplemented diet (1% by wt). In a recent review by De Crescenzo et al. [51], they looked at the impact of PUFAs on patient-important outcomes in children and adolescents with autism spectrum disorder. In their study, PUFAs were considered from fish oil containing eicosapentaenoic acid (EPA), DHA, and α-linolenic acid. This study was based on randomized controlled trials (RCTs) comparing PUFAs versus placebo or a healthy diet for the treatment of ASD in children and adolescents. The results showed that PUFAs were superior compared to the placebo in reducing anxiety in individuals with ASD but worsened quality of sleep compared to a healthy diet, both with low to very low certainty of evidence. The same review found that PUFAs were no better than the placebo in reducing aggression, hyperactivity, adaptive functioning, irritability, restricted and repetitive interests, and behaviors and communication. Findings from the De Crescenzo study [51] and others [48,49,50,52,53] were inconclusive, suggesting that omega-3 PUFA supplementation has no effect on the core aspects of ASD once the diagnosis is established.

## 4. Materials and Methods

### 4.1. Animals, Diets, Stress, and Experimental Groups

#### 4.1.1. Animals

Male homozygous serotonin transporter (SERT) knock-out (KO) mice with a C57BL6/J background and male/female C57BL6/J mice were purchased from Jackson Laboratories (Bar Harbor, ME, USA). From these mice, heterozygous SERT KO female (HT) dams and wild-type (WT) dams were generated in our laboratory. Eight-week-old experimental dams from both groups were paired with WT males and inspected for a vaginal plug the following morning. Identification of a plug was designated as gestational day 0. Additionally, Hsd:NSA(CF-1) male and female mice were purchased from Jackson Laboratories and mated to give birth to induce lactation and maternal behavior (foster dams). Within 24 h of experimental offspring birth, pups from foster dams were removed and replaced with experimental offspring. All pups were weaned on post-natal day (PD) 21, behaviorally tested after post-natal day 60 and sacrificed around post-natal day 70 for subsequent brain lipid and GABAergic related expression analysis. Animals were maintained in a temperature- and humidity-controlled room at 25 ± 2 °C on a 12-h light/dark cycle with food and water available ad libitum. All animals were housed in clear carbonate cages provided with aspen shaving bedding.

#### 4.1.2. Diet

Two weeks prior to mating, all experimental and foster dams were randomly assigned to one of two dietary groups, control diet (AIN-93G, #103619, Dyets Inc., Bethlehem, PA, USA or LabDiet 5008) and 1% DHA diet (#103598, Dyets Inc., Bethlehem, PA, USA), which they remained on throughout gestation, lactation, and offspring postweaning.

#### 4.1.3. Gene/Environment Interaction

To generate gene/environment interaction mouse models and the effects of dietary DHA supplement, experimental dams were randomly assigned to one of eight conditions represented in a 2 × 2 × 2 design of genotype × prenatal stress × diet: WT/non-stressed/AIN-93G (WNA), WT/non-stressed/DHA diet (WND), WT/stressed/AIN-93G diet (WSA), WT/stressed/DHA diet (WSD), HT/non-stressed/AIN-93G (HNA), HT/non-stressed/DHA (HND), HT/stressed/AIN-93G (HSA), and HT/stressed/DHA (HSD). The offspring from each experimental dam was also described by the same group name.

#### 4.1.4. Prenatal Chronic Variable Stress

Chronic variable stress (CVS) was administered on gestational day 6 through birth for all mice in the prenatal stress groups. Stressors included: constant light exposure (36 h), exposure to fox urine (1 h), overnight exposure to novel objects (marbles) in the home cage, restraint (10 min), overnight exposure to novel noise (radio static), and multiple cage changes during the light cycle. Each of these stressors was presented in succession over 6 days, with one stressor presented per day, and this pattern was repeated 2.5 times. Stressors were chosen on the basis that they do not cause pain and have minimal influence on food intake and weight gain [1,47,81].

This resulted in 8 groups, 8 mice per group (4 males and females) as shown in Table 1. Behavioral testing was assessed in male and female mice, selecting 2 per litter where possible to minimize litter effects. The lipid peroxidation assay was carried out using the left cortex of control and DHA-supplemented male mice. Similarly, the level of GABA expression was assessed with the right cerebral cortex and right striata.

### 4.2. Behavioral Assays

#### 4.2.1. Social Approach

To assess social preference behaviors at post-natal day (PD) 60, a three-chamber test was employed using a Plexiglas apparatus (54.5 cm × 41.5 cm × 22 cm) divided into three equal chambers (17.5 cm × 41.5 cm × 22 cm) by walls with openings (10.25 cm × 2.5 cm) allowing free movement between chambers [1,47,75,82]. Non-experimental “stranger” mice were sex and age matched C57Bl/6 to the experimental mice and prepared prior to the task as described previously [1]. Experimental mice were first habituated to the apparatus for 10 min. During the social approach phase, a stranger mouse was placed under a wire cage (Galaxy Cup, Spectrum Diversified Designs, Inc., Glenwillow, OH, USA) within one chamber, while the opposite chamber contained an empty wire cage. The experimental mouse was then allowed to explore all chambers for the following 10 min. Time spent proximal (~2 cm around the wire cage) to the stranger mouse was recorded as an indicator of social interaction. To evaluate social novelty, a second stranger mouse was introduced into the previously empty chamber, while the original stranger remained in place. The mouse’s interactions with the new stranger were recorded for an additional 10 min to assess its interest in social novelty. Sides were counterbalanced between each experimental animal as the first stranger introduction was alternated between the left and right chambers. Video tracking software was used to measure the time spent in each chamber and proximity to the strangers.

#### 4.2.2. Open Field Test

Locomotor and exploratory behavior were evaluated the following day using the open field test. Each mouse was placed individually in a 45 cm × 45 cm × 22 cm Plexiglas arena and observed for 20 min. AnyMaze software (version 7.16) tracked the total distance traveled and time spent moving. When mice were introduced to this type of apparatus, they tended to exhibit thigmotaxis behavior (i.e., staying near or following the walls) but decreasingly so over time spent on the apparatus. Thus, time not spent in the inner region of the arena was measured and used to quantify anxiety-like behaviors [83].

#### 4.2.3. Elevated Plus Maze

Anxiety-like behavior was further examined using the elevated plus maze, which consists of two open arms (35 cm × 6.25 cm) and two closed arms (35 cm × 6.25 cm × 21 cm) elevated 75 cm above the ground and connected by a central platform (5 cm × 5 cm) [84]. Mice were placed in the center of the maze and allowed to explore for 10 min. The time spent in open versus closed arms was recorded, and the open arm ratio was also used (Time spent on open arm/Total time in apparatus) as an index of anxiety.

#### 4.2.4. Repetitive Behaviors

Repetitive behavior was assessed using self-grooming and marble burying tests. Self-grooming was observed during the 20-min open field test, with the total duration and frequency of grooming bouts recorded, as excessive grooming is associated with repetitive behaviors in neuropsychiatric models [77]. Marble burying behavior was evaluated in a cage with corncob bedding. After a 20-min habituation, 20 glass marbles were arranged in a 5 × 4 grid on the bedding. Mice were allowed to interact with the marbles for 20 min, after which the number of buried marbles (more than two-thirds covered by bedding) was recorded [78,85,86].

### 4.3. Tissue Preparation

After the completion of behavioral assays (~PD70), mice were anesthetized using a ketamine/xylazine mixture and intracardially perfused with 30 mL of 0.1 M phosphate-buffered saline (PBS) containing 0.05 U/mL heparin (Sigma-Aldrich, Inc., St. Louis, MO, USA) and 2 mM ProHance (Bracco Diagnostics, a gadolinium-based contrast agent, Milan, Italy) at a rate of approximately 1 mL/min using a minipump. Tissue was then extracted, divided by hemisphere, and rapidly frozen with liquid nitrogen.

### 4.4. LC-MS/MS Analysis of 4-HHE and 4-HNE

Determination of 4-HHE and 4-HNE by LC-MS/MS analysis was performed as described by Yang et al. [59]. Briefly, the frozen left cerebral cortex was weighed and homogenized in double distilled water using a weight to water ratio of 1:8 (wt/vol). The homogenates were centrifuged at 17,000 rpm for 20 min at 4 °C, and the supernatant was collected and stored at −80 °C prior to use. The total protein concentration in the supernatant was determined using the Bicinchoninic Acid Assay (Sigma-Fisher, St. Louis, MO, USA).

The sample preparation stage involved three main steps, phospholipid removal, derivatization, and desalting, as previously described [58,59]. Briefly, an aliquot of the sample was added to an equal volume of internal standard (4-HHE-d3), and acetonitrile containing 1% formic acid was added to the mixture. Phospholipids were removed using solid-phase extraction (SPE) using a 1 mL Phree^TM^ cartridge (Phenomenex, Torrance, CA, USA). The clear eluate was dried under nitrogen and derivatized by adding freshly prepared acidified 1,3-cyclohexanedione reagent. The mixture was incubated at 60 °C for 1 h and then cooled on ice prior to desalting using a C18 SPE cartridge (Waters Corp, Milford, MA, USA). The C18 cartridge was preconditioned with methanol (0.7 mL) and equilibrated with water (0.7 mL). The cooled derivatized mixture was loaded onto the cartridge, washed twice with water (0.7 mL), and followed by 5% acetonitrile in water (0.7 mL). The analytes of interest (4-HHE, 4-HNE, and 4-HHE-d3 derivatives) were eluted with 100% acetonitrile (0.7 mL) and dried with a steady stream of nitrogen gas. The samples were reconstituted in 40% methanol in water with 0.1% formic acid (300 µL) and were ready for LC-MS/MS analysis using the Waters Acquity UHPLC system (Waters Corp, Milford, MA, USA) equipped with a quaternary solvent manager in conjunction with a C18 reversed phase column, Luna Omega 100 Å (1.6 µm × 50 × 2.1 mm, Phenomenex, Torrance, CA, USA).

### 4.5. Quantitative PCR

Offspring brains (n = 4–5 per sex, condition, and area), right cortices, and right striatum were collected. Total RNA was extracted from micro-dissected brain tissue samples using an RNeasy Mini Kit (Qiagen, Hilden, Germany). RNA concentrations were quantified using a Nanodrop Spectrophotometer (Thermo Scientific, Waltham, MA, USA). First-strand cDNA was synthesized using the Transcriptor First-Strand cDNA Synthesis Kit (Roche, Indianapolis, IN, USA). Real-time PCR was conducted on an HT-7900 (Applied Biosystems, Foster City, CA, USA) with Power SYBR Green Master Mix (Applied Biosystems) using specific primers (Integrated DNA Technologies, Coralville, IA, USA). Glutamate decarboxylase 1 (*Gad1*): CACCGAGCTGATGGCATCT, AGATCTTCAGGCCCAGTTTT; Glutamate decarboxylase 2 (*Gad2*): TTTGCGCACTCTGGAAGACA, GCTGACCATTGTGGTCCCATA; Potassium chloride cotransporter 2 (*Kcc2*): GCGGGATGCCCAGAAGTCTA, GATGCAGGCTCCAAACAGAACA; Gapdh (RNA): GGTGAAGGTCGGTGTGAACG, CTCGCTCCTGGAAGATGGTG; Gapdh (DNA): TGACGTGCCTGGAGAAAC, CCGGCATCGAAGGTGGAAGAG; Na-K-Cl cotransporter (*Nkcc1*): AGGAGCATTCAAGCACAGCTAACA, CGCTCTGATGATTCCCACGA; Parvalbumin (*Parv*): TGTCGATGACAGACGTGCTC, TTCTTCAACCCCAATCTTGC; Somatostatin (*Sst*): AGGACGAGATGAGGCTGG, CAGGAGTTAAGGAAGAGATATGGG; Reelin (*Reln*): TTACTCGCACCTTGCTGAAAT, CAGTTGCTGGTAGGAGTCAAAG; Scn2a: GGGTTGCATATGGTTTCCAA, CCCAAGGCATTTGCAGTTA; Calbinden (*Calb1*): AGATCTGGCTTCATTTCGACG, TTCATTTCCGGTGATAGCTCC.

The qPCR reactions were carried out using GeneAmp PCR Mastermix (Applied Biosystems) in either a 7900HT or StepOneTM Instrument (Applied Biosystems). The cycle threshold (CT) for each gene of interest was normalized to the CT of *Gapdh* for the same sample. The difference in cycle number (ΔCT) was normalized to male control condition and then converted to gene expression values using the following formula: Expression = 2^−ΔCT^

### 4.6. Statistical Analysis

For determination of 4-HHE and 4-HNE levels via LC-MS/MS, samples were prepared in three biological replicates and three analytical replicates for each sample. Statistical analyses were performed with GraphPad Prism (version 8.3; GraphPad Prism Software Inc., San Diego, CA, USA). The results were expressed as the mean ± standard error of the mean (SEM). Depending on the experiments, samples were analyzed by non-parametric *t*-tests or two-way ANOVA to compare among control (A) and DHA (D) diet groups of each genotype × prenatal stress combination: WT/non-stressed (WN), WT/stressed (WS), HT/non-stressed (HN), and HT/stressed (HS), here after referred to as the “mouse model”. Post hoc comparisons were carried out using Tukey HSD and a priori hypotheses were tested with pairwise *t*-tests. Differences were considered significant at *p* < 0.05 for all analyses.

Due to established male bias in the study of ASD both in prevalence (3.4:1 according to recent estimates [6]), we analyzed male and female mice separately in each behavioral test and conducted a series of two-way ANOVAs focused on the comparison and interaction of each mouse model (stressed SERT-het (HS), non-stressed SERT-het (HN), stressed wild type (WS), and non-stressed wild type (WN)) with their corresponding diet conditions (AIN control diet (A), 1% DHA (D)). For all behavioral testing analyses, males and females were conducted separately. Specific group sizes and compositions for each behavioral test are detailed in their respective results sections.

To look for changes in GABAergic system genes, a series of two-way ANOVAs were conducted to elucidate the effects of DHA supplementation by mouse model and diet; the results were run separately in each region and in each sex. Tukey post-hoc analyses were run to further define the possible relationships at work. Statistical analyses used only ddCT values, and all values are provided as the mean ± SEM for ddCT in the text, where positive values indicate downregulation. However, transformed fold change values are used for graphical depictions. Issues with RNA yields resulted in varying sample sizes per group and region. Female cortices in particular resulted in 0 usable samples for the non-stressed heterozygous SERT group for *Calb1* expression. All other genes were analyzed including the 1 sample from the HNA group in the female cortex.

## 5. Conclusions

As with our previous work [1,47,79], prenatal stress impacted social communication and repetitive behaviors, but DHA only reversed the effects of repetitive grooming events. In our previous study [59], peri-natal supplement with DHA to pregnant mothers throughout weaning pups resulted in an increase in DHA and a concomitant decrease in ARA in brain fatty acids. This feeding condition resulted in a small increase in 4-HHE but did not alter levels of 4-HNE [59]. Measurement of lipid peroxidation products in this study showed a small increase in 4-HHE in the HT group that was stressed. For unknown reasons, both the WT-fed DHA showed a significant decrease in 4-HHT and 4-HNE. Since similar changes for both 4-HHE and 4-HNE suggested possible problems with tissue processing. These effects were associated with complex, sex-specific changes in expression associated with GABAergic function. Future studies will be needed to better understand the impact of this maternal gene × prenatal stress model and partial mitigation with DHA, including examining effects on regional neuroanatomical and neurochemical effects and sex-specific effects at critical developmental windows.

## Figures and Tables

**Table 1:** Summary of experimental groups. The numbers in parenthesis represent the dams, while the other value represents the number of offspring.

	Stressed DHA HT(HSD)		Stressed AIN HT (HAS)		Stressed DHA WT (WSD)		Stressed AIN WT (WSA)	
	M	F	M	F	M	F	M	F
**Behavior**	14 (7)	14 (8)	18 (7)	15 (6)	6 (3)	5(3)	10 (5)	11 (6)
**Brain & Tissue**	4 (3)	4 (3)	4 (2)	4 (2)	4 (3)	4 (2)	4 (3)	4 (3)
	**Non-stressed DHA HT (HND)**		**Non-stressed AIN HT (HNA)**		**Non-stressed DHA WT (WND)**		**Non-stressed AIN WT (WNA)**	
	**M**	**F**	**M**	**F**	**M**	**F**	**M**	**F**
**Behavior**	17 (7)	12 (7)	8 (5)	8 (5)	10 (4)	7 (4)	6 (4)	6 (4)
**Brain & Tissue**	4 (4)	4 (4)	4 (3)	4 (3)	4 (4)	4 (3)	4 (4)	4 (4)

## Data Availability

Data available upon request. Data not available due to feasibility issues.
